# Chondrogenesis of Human Infrapatellar Fat Pad Stem Cells on Acellular Dermal Matrix

**DOI:** 10.3389/fsurg.2016.00003

**Published:** 2016-01-26

**Authors:** Ken Ye, Kathy Traianedes, Peter F. M. Choong, Damian E. Myers

**Affiliations:** ^1^Department of Surgery, St Vincent’s Hospital Melbourne, The University of Melbourne, Fitzroy, VIC, Australia; ^2^Department of Orthopaedics, St Vincent’s Hospital Melbourne, Fitzroy, VIC, Australia; ^3^Department of Clinical Neurosciences, St Vincent’s Hospital Melbourne, Fitzroy, VIC, Australia; ^4^Department of Medicine, St Vincent’s Hospital Melbourne, The University of Melbourne, Fitzroy, VIC, Australia

**Keywords:** tissue engineering, regeneration, stem cells, extracellular matrix, AlloDerm, chondrogenesis, cartilage

## Abstract

Acellular dermal matrix (ADM) has been in clinical use for decades in numerous surgical applications. The ability for ADM to promote cellular repopulation, revascularisation and tissue regeneration is well documented. Adipose stem cells have the ability to differentiate into mesenchymal tissue types, including bone and cartilage. The aim of this study was to investigate the potential interaction between ADM and adipose stem cells *in vitro* using TGFβ3 and BMP6. Human infrapatellar fat pad-derived adipose stem cells (IPFP-ASC) were cultured with ADM derived from rat dermis in chondrogenic (TGFβ3 and BMP6) medium *in vitro* for 2 and 4 weeks. Histology, qPCR, and immunohistochemistry were performed to assess for markers of chondrogenesis (collagen Type II, SOX9 and proteoglycans). At 4 weeks, cell-scaffold constructs displayed cellular changes consistent with chondrogenesis, with evidence of stratification of cell layers and development of a hyaline-like cartilage layer superficially, which stained positively for collagen Type II and proteoglycans. Significant cell–matrix interaction was seen between the cartilage layer and the ADM itself with seamless integration between each layer. Real time qPCR showed significantly increased COL2A1, SOX9, and ACAN gene expression over 4 weeks when compared to control. COL1A2 gene expression remained unchanged over 4 weeks. We believe that the principles that make ADM versatile and successful for tissue regeneration are applicable to cartilage regeneration. This study demonstrates *in vitro* the ability for IPFP-ASCs to undergo chondrogenesis, infiltrate, and interact with ADM. These outcomes serve as a platform for *in vivo* modelling of ADM for cartilage repair.

## Introduction

Cartilage lesions are common, reported in over 60% of all arthroscopic procedures, and often cause pain and disability for patients (1, 2). Cartilage damage can also predispose to further cartilage loss and development of osteoarthritis (3). The compounding issue is that articular cartilage has limited capacity for self-regeneration and healing due to its avascular nature. Current methods of osteochondral repair such as microfracture, osteochondral grafting, and autologous chondrocyte implantation have suboptimal long-term results due to formation of fibrocartilage, along with other complications such as donor-site morbidity and hypertrophy (4–8). Tissue engineering approaches, therefore, may offer treatment options that can overcome current limitations. The optimal combination of cells, scaffold, and biochemical factors may eventually result in true cartilage regeneration.

Acellular biological scaffolds have been used in many tissue engineering applications for regenerative tissue repair. Acellular dermal matrix (ADM) is one type of acellular biological scaffold. As described by Livesey et al. (9) in a proof-of-principle study in minipigs, the porcine allograft ADM was used as a full-thickness skin graft in combination with an ultrathin epidermal graft. The unique decellularisation/freeze-drying process preserves extracellular matrix (ECM) structure without causing an inflammatory or tissue rejection response when transplanted (9). The transplanted graft shows revascularisation, repopulation by host cells, and volume persistence (9). Since this early preclinical study, the human equivalent (AlloDerm^®^) has been used in numerous surgical applications including complex abdominal wall hernia repair, full thickness dermal replacement, head and neck reconstructive applications, soft-tissue defect augmentation, rhinoplasty, alloplastic breast reconstruction, vaginal repair, and tendon repair (10).

Adult mesenchymal progenitor cells can be derived from bone marrow, fat, skin, muscle, periosteum, or cord blood (11–16). Adipose tissue is an attractive source of progenitor cells due to ease of accessibility, great abundance, and chondrogenic potential (17, 18). While most adipose stromal cells have been extracted from liposuction material, a number of studies have utilized adipose stromal cells derived from infrapatellar fat pad (IPFP-ASC) (19–23) for the purposes of cartilage repair. Previous research has shown that IPFP express superficial zone protein (lubricin), which reduces the coefficient of friction at the articular surface (21). Recently, we have shown through microarray analysis of isolated IPFP-ASCs that a number of chondrogenic genes are significantly upregulated when stimulated with the growth factors TGFβ3 and BMP6 (24). We have also shown the ability for these cells to adhere and undergo chondrogenesis on a 3D printed chitosan scaffold (25).

Therefore, the aim of this study was to investigate the potential interaction between a complete biological scaffold, ADM, and stem cells *in vitro* using our previously reported approach using IPFP-ASCs in combination with chondrogenic growth facts, TGFβ3 and BMP6 (24, 25).

## Materials and Methods

### Ethics Approval and Patient Selection

Human infrapatellar fat pads were obtained intraoperatively from total knee arthroplasties after informed consent and approval from Human Research Ethics Committee at St Vincent’s Hospital (Melbourne). All necessary ethics protocols were adhered to in the process of tissue harvest and use. Only patients with primary osteoarthritis were selected. Patients with inflammatory arthritis and with a history of prior knee surgery were excluded from selection.

### Cell Isolation, Culture, and Characterization

The IPFP were harvested from three individual patients. Each IPFP was processed separately and cells from each patient were never combined in the *in vitro* culture process. The IPFP was immediately placed in sterile phosphate buffered saline (PBS) (GIBCO, Life Technologies Corporation, Carlsbad, CA, USA) and processed within 30 min of harvest. Initially, the tissue was washed several times with PBS, to remove contaminating blood. The excised fat pad was cleaned from any other adherent tissues prior to the cell isolation procedure. The subsequent remaining fat tissue was diced and digested with 0.2% Collagenase Type 1 (Worthington Biochemical Corporation, Lakewood, NJ, USA) for 3 h at 37°C under constant agitation. The entire digest was filtered through a 100-μm nylon mesh and centrifuged at 400 *g* at room temperature for 5 min to remove adipocytes (floating). The cell pellet was resuspended in Red Cell Lysis Buffer (Sigma-Aldrich, St. Louis, MO, USA) and incubated at room temperature for 10 min. The cells were then filtered through a 40-μm nylon mesh before centrifugation at 400 *g* at room temperature for 5 min. The cells were resuspended in PBS, counted, and plated in monolayer culture [75-cm^2^ tissue culture flask (Corning Inc., NY, USA)] at 5 × 10^3^cells/cm^2^ in stromal media (SM)-containing DMEM (Sigma-Aldrich, St. Louis, MO, USA) supplemented with 10% FBS (GIBCO), 1× antibiotic/antimycotic solution (GIBCO), and 1× Glutamax (GIBCO). Cultures were maintained for 48 h at 37°C in 5% CO_2_ in air (Air Liquide Australia Ltd., Melbourne, VIC, Australia). The cells were washed and media were replaced with expansion media (EM) containing stromal media with 5 ng/ml human epidermal growth factor (hEGF) (R&D Systems, Inc., Minneapolis, MN, USA) and 1 ng/ml human fibroblastic growth factor (hFGF) (R&D Systems, Inc.). The cells were cultured until 80% confluency and then harvested with 0.1% EDTA/0.25% trypsin (Sigma-Aldrich) and made into a single cell suspension for seeding onto the ADM scaffold (see below).

Cells were characterized, using flow cytometry, and a panel of known mesenchymal cell markers, other cell markers, and Ig Controls. The methods have been previously published by our group (24). Briefly, cells were resuspended in 0.5% bovine serum albumin in PBS at a final concentration of 1 × 10^6^ cells/ml. Cells aliquots (0.25 × 10^6^ cells/200 μl per tube) were stained with mesenchymal cell markers (CD29, CD44, CD73, CD90, and CD105), as well as other cell markers (CD31, HLADR, CD45) (BD Pharmingen) and IgG1 and IgG2a isotype controls (BD Pharmingen). All tubes were analyzed within 2 h of staining using an FACS Canto flow cytometer system (BD).

### Scaffold Preparation

Fresh rat skin was harvested from nu/nu rats (Australian Research Centre, Perth, WA, Australia). The method of decellularisation was slightly modified from the process previously described (9). Briefly, fresh tissue was placed immediately in RPMI 1640 medium (GIBCO) supplemented with antibiotics, followed by aseptic processing (U.S. Patent 5,336,616) performed in a biosafety cabinet. This processing involved incubating the skin in high salt solution, shaking overnight at room temperature. The epidermis separated from the dermis in this process, dermal cells were removed by incubation in non-denaturing detergent, shaking overnight at room temperature. The material was cyroprotected in a carbohydrate complex solution. The cyroprotected matrix was packed in sterile poly-Tyvek pouches (Beacon Converters Inc., Saddle Brook, NJ, USA), heat sealed then freeze-dried (Virtis Genesis 25L freeze-dryer, SP Industries Warminster, PA, USA). Freeze-dried tissue was subsequently packed and sealed in foil pouches (Beacon Converters Inc.) and stored at −80°C until use. Scaffolds were characterized histologically and immunohistochemistry for structural integrity and compared to fresh tissue. Matrix samples were pre-qualified for biological integrity using a sub-dermal implant in immunocompetent rats (not shown).

### Chondrogenic Differentiation and Culture

Confluent passage three IPFP-ASCs were harvested, counted, and resuspended in chondrogenic medium (CM) consisting of DMEM-high glucose, 1% FBS, 1% ITS, 100 nM Dexamethasone, 50 ug/ml ascorbic acid, 1× antibiotic/antimycotic, 10 ng/ml TGFβ3, and 10 ng/ml BMP6. Scaffolds were cut using a 6-mm biopsy punch (Kai Medical, Honolulu, HI, USA) and placed in a 24-Transwell^®^ tissue culture plate well inserts (6.5 mm ID, 3.0 μm pore size) (Corning Inc.) and then seeded with 7.5 × 10^5^ ASCs. Cell-ADM constructs were incubated at 37°C in 5% CO_2_ for 14 and 28 days (2 and 4 weeks) and media changed three times per week. Cell-matrix constructs in media without growth factors served as the negative control (non-chondrogenic). A total of six scaffolds for the 2- and 4-week time points for each biological sample (*N* = 3 IPFP sources; *n* = 36 wells total). These cell-ADM constructs were used for gene expression analysis. Two scaffolds cultured for 4 weeks, were used for histological and immunohistochemical analysis.

### Histology and Immunohistochemistry

After 4 weeks of culture, cell-ADM constructs were harvested, fixed overnight in 10% neutral buffered formalin (NBF) (Sigma-Aldrich) and were then paraffin embedded (Pathology Department, St Vincent’s Hospital, Melbourne) for subsequent histological and immunohistochemical analysis. Samples were sectioned (4 μm thick) and dried overnight at 37°C. Sections were deparaffinized, rehydrated through graded ethanol, and stained with hematoxylin & eosin (H&E) and toluidine blue (TB) (Sigma-Aldrich). TB staining was used to stain for proteoglycans within the cartilaginous tissue.

Accumulation of collagen Type I and II was assessed by immunohistochemistry. Briefly, sections were treated with 0.3% hydrogen peroxide (H_2_O_2_) (Merck Millipore, Darmstadt, Germany) for 5 min, subjected to Proteinase K for antigen retrieval (Dako, Glostrup, Denmark) for 4 min and were blocked using 10% normal rabbit serum (NRS) (Dako) for 30 min at room temperature. These sections were incubated with the following primary antibodies: mouse monoclonal anti-human Type II collagen antibody (1:500) (MP Biomedical, Solon, OH, USA), goat polyclonal anti-human Type I collagen (1:500) (SouthernBiotech, Birmingham, AL, USA), and mouse monoclonal anti-human cartilage proteoglycan antibody (1:500) (Merck Millipore, Billerica, MA, USA) for 60 min at 37°C. Isotype negative controls were used at the same concentration as their respective primary antibodies: Goat IgG isotype control (SouthernBiotech) and mouse IgG isotype control (Invitrogen, Life Technologies Corporation). Secondary antibodies used were biotinylated rabbit polyclonal anti-goat and rabbit anti-mouse antibodies (Dako). Secondary antibodies were applied for 30 min at room temperature followed by horseradish peroxidase (HRP)-conjugated streptavidin using the Vectastain ABC kit according to the manufacturer’s instructions (Vector Laboratories Burlingame, CA, USA). The reaction was developed using peroxidase substrate 3,3-diaminobenzidine (DAB) for 5 min (Dako). Sections were counterstained with hematoxylin, dehydrated, cleared, and mounted with Pertex (Histolab Products AB, Gothenburg, Sweden). Fresh rat skin and rat ADM were stained using the same protocols to serve as controls.

### Quantitative Real Time PCR

Two and four week cell-ADM constructs were pulverized in liquid nitrogen using a mortar and pestle and then homogenized in 1 ml of Trizol solution (Ambion, Life Technologies, Carlsbad, CA, USA). Samples were purified using the Trizol method and silica membrane-based commercial extraction kit (RNeasy mini kit, QIAGEN Pty Ltd., Hilden, Germany) according to the manufacturer’s protocol. RNA from pre-differentiated cells (day 0) was also extracted. The RNA concentration and purity were measured using the Agilent 2100 BioAnalyzer (Agilent Technologies, Santa Clara, CA, USA). Complimentary DNA copies were reverse transcribed from 200 ng total RNA for all samples using oligo-dT primers and omniscript reverse transcriptase kit according to the recommendations of the manufacturer (Qiagen Pty Ltd.). Quantitative PCR (qPCR) was performed using standard TaqMan^®^ Probe-Based Gene Expression Analysis protocols using commercial available probes for Collagen Types I and II, SOX 9, and Aggrecan (Invitrogen, Life Technologies Corporation). The Taqman primer ID for each gene was as follows: *COL1A2* (Hs00164099_m1), *COL2A1* (Hs00264051_m1), *SOX9* (Hs01165814_m1), and *ACAN* (Hs00153936_m1). *GAPDH* was used as the housekeeping gene for relative quantification of gene expression (Hs02758991_g1). Liquid handling was performed by the CAS1200 series robot by Corbett Robotics (Corbett Life Sciences, Qiagen, Hilden, Germany). Subsequent PCR reaction was performed using the Roche Lightcycler 480 (Roche, Basel, Switzerland) and preliminary data analysis performed using the Lightcycler 480 software version 1.5 (Roche).

### Data Analysis

All numerical data analysis of relative quantification of qPCR results was performed in Microsoft Excel 2010 (Microsoft Corp., CA, USA) and GraphPad Prism 6.0 (GraphPad Software, La Jolla, CA, USA) using the 2^(−ΔCT)^ method. Means, SD, SEM, and 95% confidence limits were calculated for each set of results. Friedman’s test was used to assess significance between multiple sets of data.

## Results

### Cells and Material Characterization

Infrapatellar fat pad-derived adipose stem cells showed a typical spindle-shaped fibroblastic morphology, staining strongly for CD29, CD44, CD73, CD90, and CD 105 (MSC markers). Staining for CD31 (endothelial cell marker), HLADR (hematopoietic cell marker), and CD 45 (leukocytic cell marker) and the isotype controls (IgG1 and IgG2a) were negative, as per our previously published data (24).

**Figure 1:**
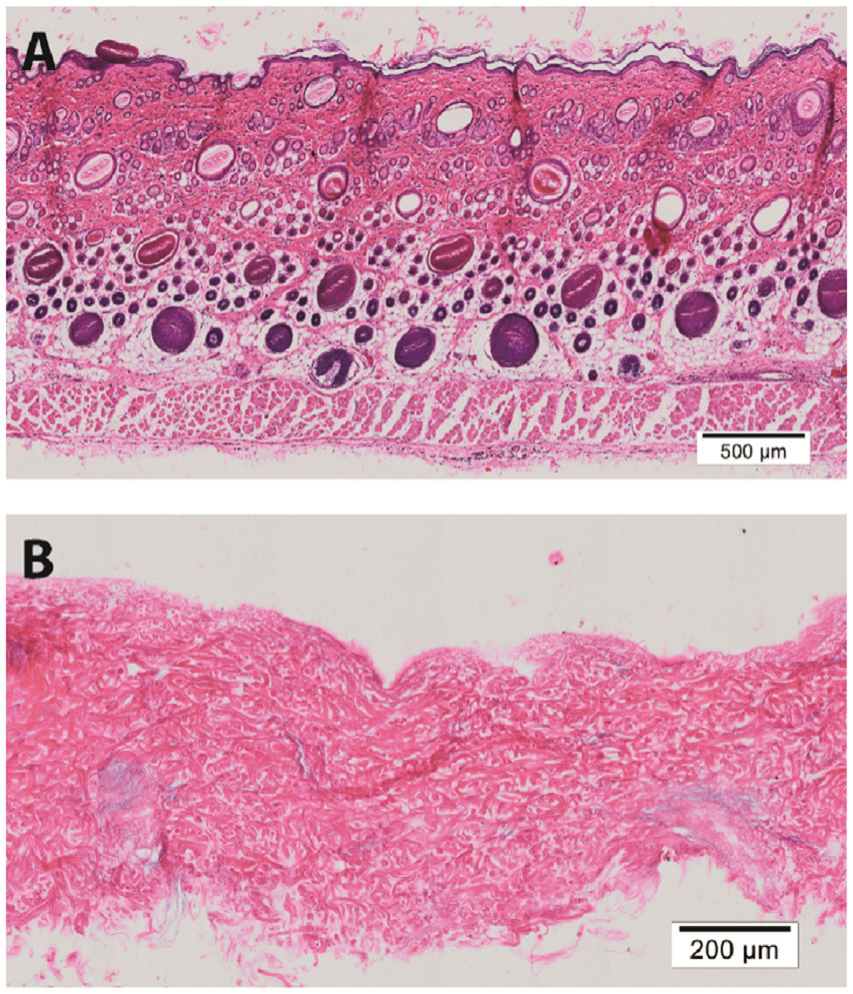
**Histology of processed rat skin**. Representative figures of H&E of full thickness rat skin **(A)** and a preparation of rat acellular dermal matrix (ADM) **(B)**. This rat ADM preparation shows the removal of the epidermis and lack of cells in the dermal layer. The histoarchitecture of the dermis of ADM is comparable to fresh skin. Scale bars as indicated. Magnification: 10×.

**Figure 2:**
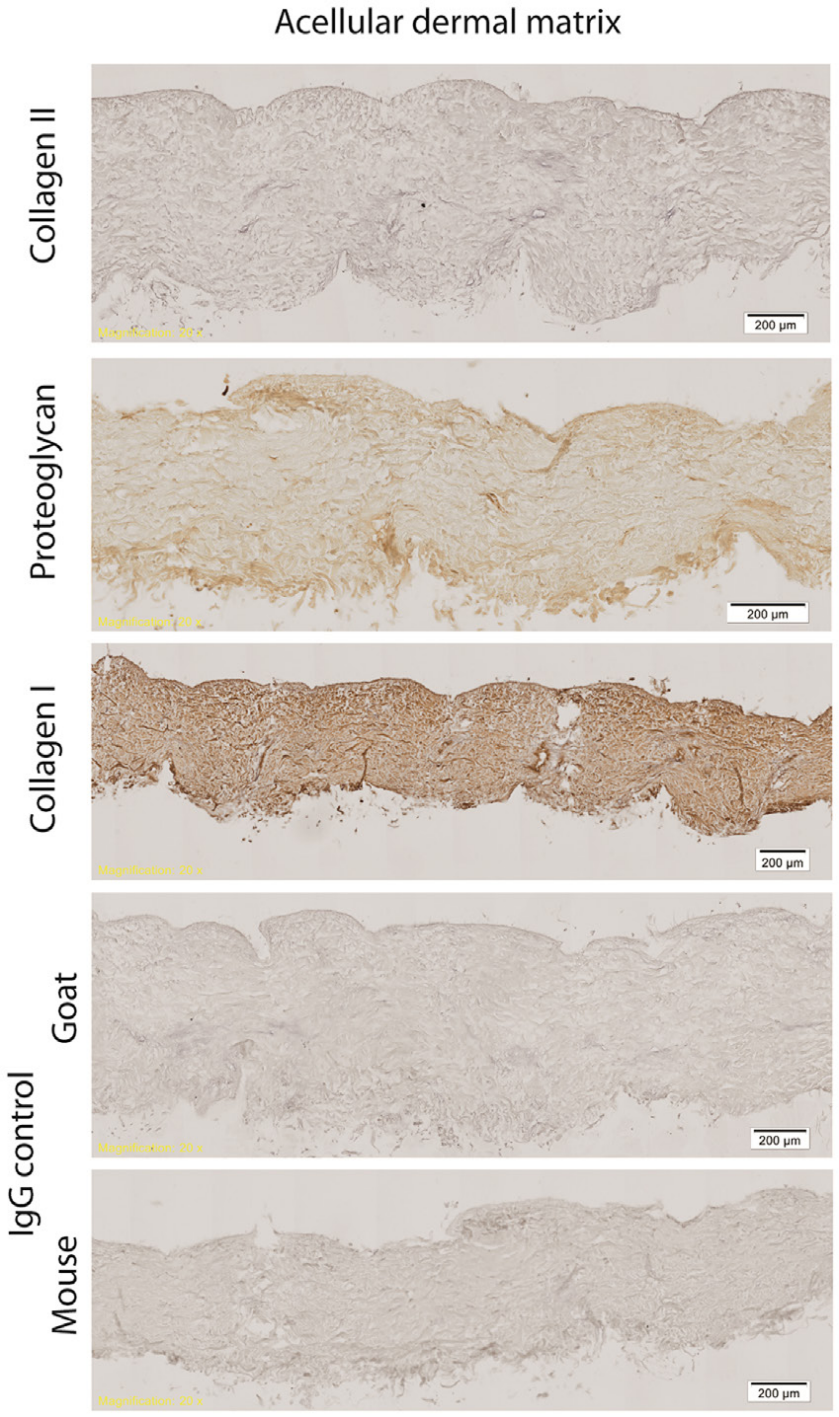
**Immunohistochemistry of processed rat skin**. Representative figures of immunohistochemistry of rat ADM shows intact bundles of collagen Type I fibers throughout the matrix. Some minor proteoglycan staining is evident throughout the ADM. There is no staining for collagen Type II. All isotype controls (IgG mouse and goat) were negative. Scale bars as indicated. Magnification: 10×.

Hematoxylin & eosin staining of normal rat skin and acellular rat dermis shows the absence of the epidermal layer in the rat ADM, no cells present and normal histoarchitecture of the collagen fibers within the dermis (Figure 1). Immunohistochemical staining for collagen Type I revealed intact bundles of collagen Type I fibers throughout the matrix as expected. There is no staining for collagen Type II as expected. IgG isotype control stains for both mouse and goat serve as negative controls for the immunohistochemistry stains (Figure 2). Subdermal implants into immunocompetent rats showed engraftment, revascularization, repopulation of rat ADM with host cells, and no indication of any inflammatory response or fibrous encapsulation of the tissue. These results indicated that the rat ADM was processed appropriately (results not shown).

### Histology and Immunohistochemistry of Chondrogenic Cultures

Hematoxylin & eosin staining of week 4 *in vitro* chondrogenic cell-ADM constructs, showed a change in the overall shape of the construct compared with the control. While the control remained flat, the chondrogenic culture had developed a cap of cartilaginous tissue, changing the ADM, which was soft and was not tethered within the well. There was evidence of stratification of cell layers within this *in vitro* construct, with the development of a hyaline-like cartilage layer superficially. Cells within the cartilaginous cap displayed chondrocytic morphology, namely large, rounded cells encapsulated in lacunae. There was also evidence of cellular infiltration within the ADM, which did not display chondrocytic morphology and remained fibroblastic in their appearance. A mixed population of cells was seen within the “ADM-cell interaction zone” between the cartilage cap and the ADM, indicating significant cell-matrix interaction had occurred.

**Figure 3:**
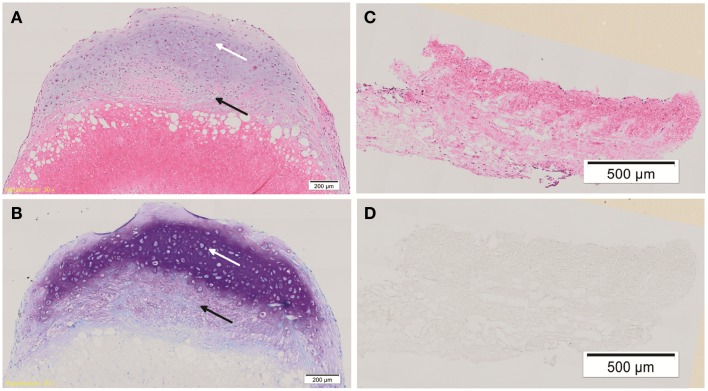
**Histology of cell-ADM constructs**. Representative histological figures of H&E and toluidine blue (TB) staining are shown for week 4 chondrogenic **(A,B)** and control **(C,D)** IPFP-ASCs on ADM. Chondrogenic cell-ADM constructs show cells within the cartilaginous cap displaying chondrocytic morphology and strong staining for TB indicative of the presence of proteoglycans (white arrow). There is also cellular infiltration within the ADM. Cells of a mixed morphology can be seen within the “ADM-cell interaction zone” (black arrow). No evidence of chondrogenic features could be detected in the control group. Scale bars as indicated. Magnification: 10×.

Toluidine blue staining was positive for proteoglycans especially toward the center of the cartilaginous cap compared with the upper, superficial layer. There was weaker staining within the “ADM-cell interaction zone,” and no staining within the ADM itself. The control constructs did not stain for toluidine blue (Figure 3).

**Figure 4:**
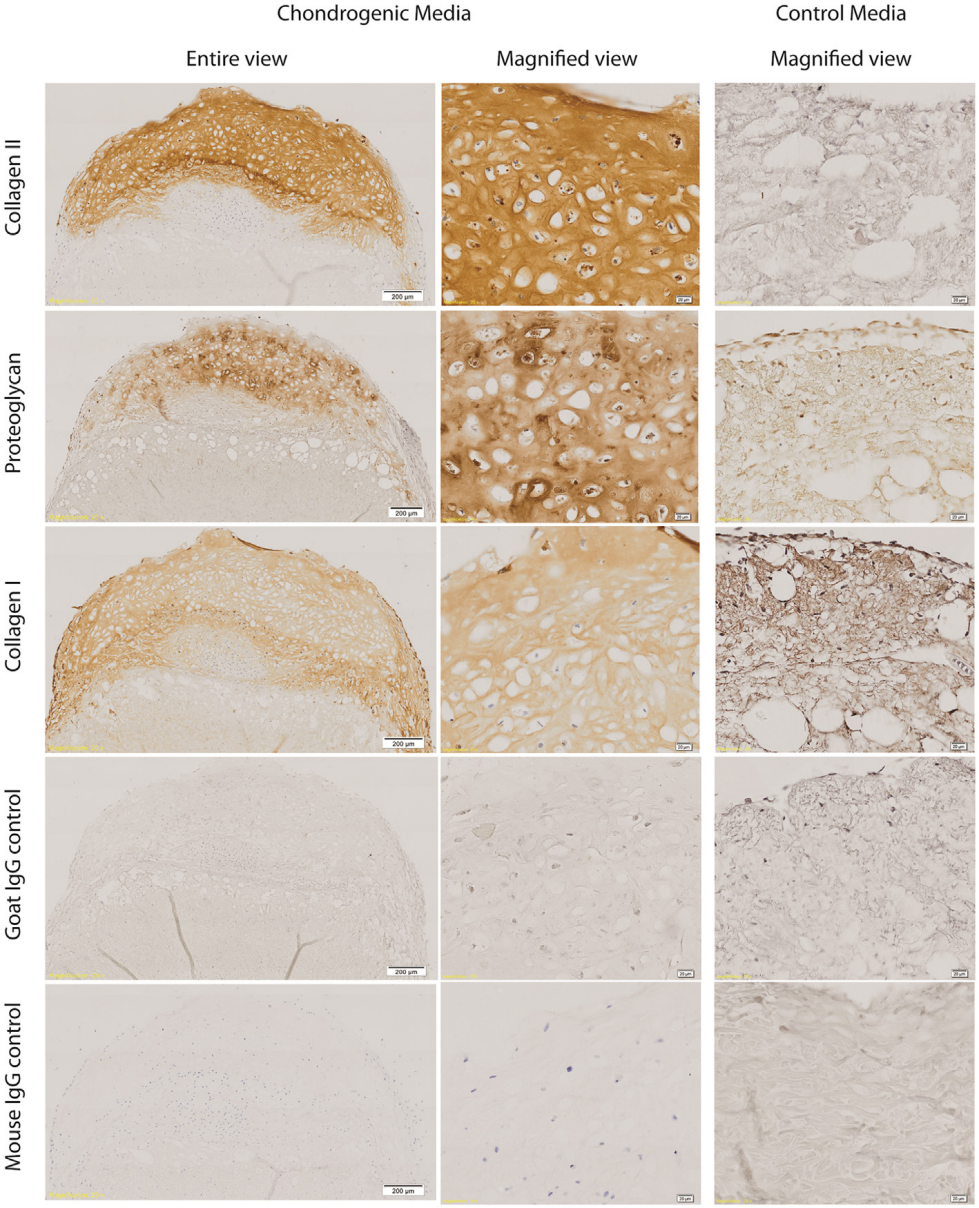
**Immunohistochemistry of cell-ADM constructs**. Representative figures of immunohistochemical staining of week 4 chondrogenic and control IPFP-ASCs–ADM constructs for collagen Type II and proteoglycan staining of the cartilaginous cap. There was diminished collagen Type II staining within the zone of interaction and no staining within the ADM. Collagen Type I was detected throughout, as well as within the ADM. However, there was weaker collagen Type I staining centrally within the cartilaginous cap compared with the periphery and the ADM-cell interaction zone. All isotype control staining were negative. Scale bars as indicated. Magnification: 10×.

Type II collagen was detected in the ECM of the cartilaginous cap, surrounding the cartilaginous cells. There was diminished Type II collagen detected within the zone of interaction and none detected within the ADM. Collagen Type II was not detected in any control samples. Similarly proteoglycan was detected in the ECM of the cartilaginous cap with decreased expression in the zone of interaction and the ADM. There was weak antibody staining of proteoglycans within the control ADM, which prompted questions of non-specific binding of the antibody or detection of non-cartilage specific proteoglycans within the ADM. Some proteoglycans may remain within the ADM even after decellularisation. Some collagen Type I was detected within the cartilaginous cap; however, greater intensity was seen around the periphery of the construct and within the ADM-cell interaction zone, and less so within the center of the cartilaginous cap. Collagen Type I was also seen in the ADM itself as well as the control construct as expected. All isotype control staining was negative (Figure 4). While the “ADM-cell interaction zone” layer had minimal staining for collagen Type II and proteoglycans, cells continued to be grouped or clustered, with collagen fibers of the ADM interspersed more readily within this layer, forming what appears to be a transition from a predominantly cartilaginous upper zone to the ADM biomaterial below.

### Gene Expression

**Figure 5:**
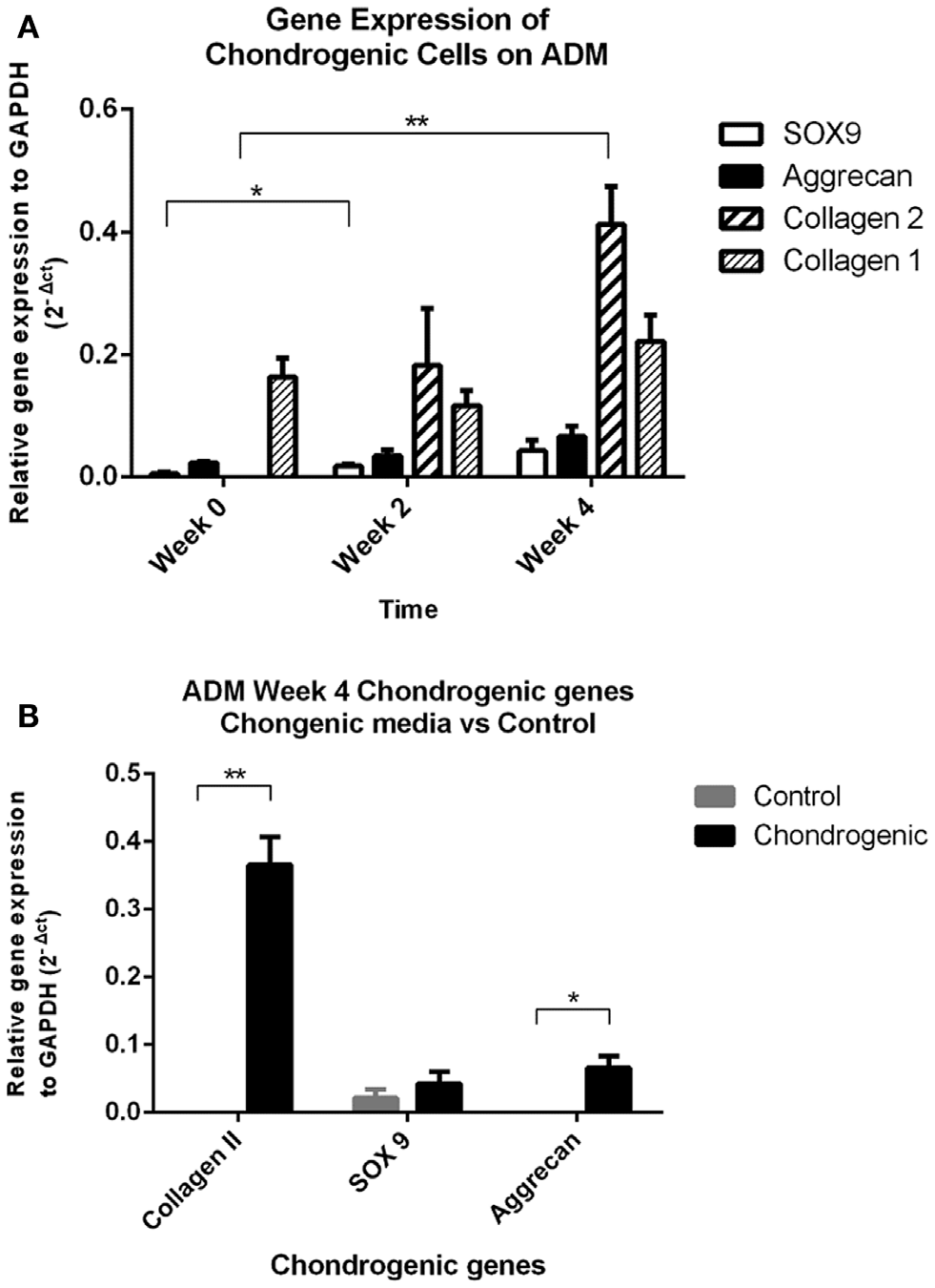
**Quantitative PCR results**. **(A)** Real time qPCR data comparing changes in mRNA gene expression (relative to GAPDH housekeeping gene) in the chondrogenic group showed significant increases in collagen Type II and SOX9 gene expression over the 4-week period. Collagen Type I was expressed by the cells (day 0), and levels remained unchanged over the 4 week period. **p < 0.005 Friedman’s test, *p < 0.05 *t*-test. **(B)** Real-time qPCR data comparing gene expression relative to GAPDH between the chondrogenic group and control group at the 4-week time point. Both collagen Type II and aggrecan gene expressions were significantly increased by week 4 in the chondrogenic group compared to the control group. Collagen Type II gene expression was undetectable in the control group at week 4. **p < 0.005 Friedman’s test, *p < 0.05 *t*-test.

All mRNA expression of chondrogenic markers (*COL2A1*, *SOX9*, *ACAN*) were increased in the chondrogenic constructs from week 0 to week 4. There were undetectable levels of *COL2A1* gene expression in the IPFL-ASCs prior to plating. In contrast, collagen Type II expression was present at 2 weeks and increased significantly by week 4 (*p* < 0.005, Friedman’s test). The expression of *SOX9* and *ACAN* increased over the 4 weeks. However, only the increase of *SOX9* expression from week 0 to week 2 was statistically significant (*p* < 0.05, *t*-test). Collagen Type I (*COL1A2*) gene expression was present in the cells from the outset and there was no significant change by week 4 (Figure 5A). After 4 weeks, *COL2A1* expression was undetectable in the control group and only low levels of *SOX9* were expressed in the control. The expression of *COL2A1* and *ACAN* at week 4 was significantly greater in the chondrogenic group compared with the control group (*p* < 0.05) (Figure 5B). These results are consistent with the development of cartilage-like material *in vitro*.

## Discussion

The clinical dilemma of cartilage repair after injury remains unsolved. The natural course of osteochondral defects toward fibrocartilage repair is essentially scar formation of the articular surface. The resulting fibrocartilaginous repair consists of predominantly collagen Type I, does not resemble hyaline cartilage in its biochemical and biomechanical properties, and leads to early degeneration and potentially early osteoarthritis (5). To achieve true regeneration without scar, many researchers have looked at acellular biological scaffolds. Decellularised tissues act as natural scaffolds comprising of extracellular matrix with varying degrees of structural collagen and carbohydrate preservation. The theory behind this approach is that the residual material should provide the perfect natural environment for cellular reattachment, migration, differentiation, and proliferation, to achieve regeneration against scar formation (26). Recently decellularised biological matrices have been used as a substrate for regeneration of a variety of tissue types including skin, cartilage, bladder, spinal cord, and myocardium (27–31). Acellular cartilage matrix has been used for treatment of chondral and osteochondral defects using various methods of scaffold preparation (32–34). A common issue with decellularised cartilage tissue is the loss of biomechanical strength (35). However, a report by Kang et al. (36) shows, in a rabbit osteochondral model using rabbit adipose tissue (dorsal nuchal) stem cells with cross-linked human acellular cartilage matrix, that the repair tissue was approximately 80% in mechanical, GAG, and collagen type II composition compared to native cartilage at 6 months. However, integration of the implant with the host cartilage continues to be an issue (36).

In previous work, we have reported the potential for IPFP-ASCs to undergo chondrogenesis on a 3D printed chitosan scaffold (25). The present study further demonstrates the versatility of IPFP-ASCs to undergo chondrogenesis on a biologically derived substrate such as ADM. However, the IPFP-ASCs were observed to interact differently with ADM, which is an intact biological matrix. The formation of what appears to be layers of tissue, each with unique features in terms of cellular morphology, extracellular matrix staining, and distribution of collagen Types I and II Type is evident. The integration between these layers is also apparent. These outcomes highlight the potential of ADM as a cell-delivery vehicle with the potential to promote integration and formation of lamellar structures. The cells are able to attach, proliferate and differentiate while interacting with the ADM. This may aid cartilage repair *in vivo*, by acting as a substrate that allows both stem cells and host tissue (bone and cartilage) to integrate seamlessly and repair the tissue.

Within the cartilaginous layer, further differentiation of staining can be seen. Cells on the surface appear to be aligned more horizontally, with weaker staining for proteoglycans as evidenced by the toluidine blue and greater staining for collagen Type I compared with staining in the center of the cartilaginous layer. This has similarities with the distribution of collagen Type I collagen and proteoglycans within mature hyaline cartilage. The significance of this phenomenon is limited in the *in vitro* setting, and further investigation is warranted under *in vivo* conditions such as, seen with mechanical pressure or bioreactors (37).

Since collagen Type II staining in the ADM control tissue is absent, all collagen Type II produced can be attributed to the differentiation of IPFP-ASCs into a chondrocytic phenotype. Histologically, the co-localization of collagen Type II and Type I may provide evidence of early developmental progression at 4 weeks *in vitro*. Collagen Type I is also expressed in early chondrogenesis as part of the transformation that occurs from mesenchymal cells to chondrocytes, consistent with the pre-natal development of the knee joint, which starts with a condensation of the mesenchyme between the two long bones prior to the distinct development of the articular surfaces of the long bones (38). In this study, constructs were maintained for only 4 weeks and may indicate the need to extend the time period for further clarification of the *in vitro* development sequence. Changes to the composition and structure of the scaffold over time may also impact the production of collagen Type I in the cells. Since this was an *in vitro study*, it is difficult to speculate how much remodeling may occur in the *in vivo* setting and hence, potential progression toward a normal hyaline cartilage structure, or complete degradation of the constructs. Our group is currently working toward a full *in vivo* study to evaluate the ADM-cell cartilage constructs.

Proteoglycan staining in ADM tissue is to be expected. Some level of proteoglycans remains even after the decellularisation process (39). These proteoglycans may possess functional properties that promote cell growth regulation, growth factor binding, and immunoregulation (40, 41) and have been shown to directly interact with growth factors such as TGFβ, TNFα, PDGF, EGFR, and ILGFR (42–45). The presence of proteoglycans within the ADM may contribute to the cellular infiltration, growth and differentiation of IPFP-ASCs in the presence of our chondrogenic growth medium (TGFβ3 and BMP6). Pietramaggiori et al. (46) showed that AlloDerm (human ADM) was able to bind approximately 50% of growth factors (TGFβ, PDGF, VEGF, and EGF) using platelet-rich plasma (PRP) thereby providing a growth factor delivery vehicle *in vivo* (46).

In conclusion, it is apparent that IPFP-ASCs appear to provide an excellent source of cells for chondrogenesis. While human ADM has been used extensively in the clinical setting for diverse surgical procedures, its use in cartilage repair is novel. We believe the principles which make ADM versatile and successful for tissue regeneration are applicable to cartilage regeneration. This study is the first to demonstrate, in an *in vitro* setting, the ability for IPFP-ASCs to undergo chondrogenesis, infiltrate and interact with the ADM material. These outcomes serve as a platform for moving to *in vivo* modeling of ADM for cartilage repair.

## Author Contributions

All authors contributed to the design and planning of this study. The majority of the laboratory work was conducted by KY, and supervised and assisted by KT and DM. The funding for this project primary came from work done by PC and DM. The paper was written by KY with revisions made by KT, DM, and PC.

## Conflict of Interest Statement

The authors have no other relevant affiliations or financial involvement with any organization or entity with a financial interest in or financial conflict with the subject matter or materials discussed in the manuscript apart from those disclosed. No writing assistance was utilized in the production of this manuscript.
